# Dental Jottings

**Published:** 1872-02

**Authors:** H. L. Sage


### Communications.

### DENTAL JOTTINGS.

BY. H. L. SAGE.

*Dental Irregularities.*

Perhaps there is nothing in which are combined in part the principles and practice of both operative and mechanical dentistry, requiring more time, care and patience on the part of those engaged, than the correction of an irregular denture—especially if a complicated case, in which the adaptation and change of appliances must be frequent, and the teeth to be moved numerous, or the deformity considerable.

To adopt those methods by which we may the best compass the end in view, when cases are numerous and diversified, may require thought—sometimes, perhaps, investigation and experiment—methods or appliances suitable for one class of irregularities not being applicable to, or efficient in, another, depending on location in the inferior or superior maxilla, extent, antagonism, etc., etc.

When, as is sometimes the case, such irregularities are taken in hand before the patient has reached that period of life in which he is supposed to fully possess those qualities of endurance, perseverance and firmness which so much add to the probabilities, or give assurance of success, the difficulties to be encountered are readily apparent. Such are, indeed, "labors of love"—and most will doubtless be disappointed, who expect to reap therefrom pecuniary profit. But, as a school for the exercise of the essential, manly, and Christian virtues and qualifications—such as patience, experience, hope, etc., and as a means of improvement for the inventive faculties, they are certainly equal, if not superior, to many which the dentist is called upon to treat. Days, weeks, or months, he must "learn to labor and to wait," satisfied if he make but slow progress—and, at the same time, by encouraging words must he reassure the young, timid—perhaps nervous and discouraged patient—so as to insure that co-operation which is so necessary to a successful result.

Herewith is presented a description of an irregularity which may be taken as the representative of a class, not rarely presented, it is true, but which may involve methods of practice somewhat different from those usually employed—perhaps not.

James B., aged thirteen, appeared for the correction of an irregular denture, the teeth presenting the following conditions: The right cuspidatus was much projected anteriorly and obliquely, with about one half of the crown erupted. The lateral incisor of the same side appeared inside of the arch, back of the cuspidatus and central incisor—its corresponding under tooth shutting outside of it. The left permanent cuspidatus had not pierced the gum when the correction was commenced, its temporary predecessor being in place—which, being extracted, allowed the permanent to cut through during the process of regulating, and which had attained to nearly full size on completion of the operation.

The left central incisor occupied a position irregularly, standing obliquely to the arch, having the appearance of being turned one-quarter around on its axis.

Not only were the foregoing conditions presented, but the gums over the central and the left lateral incisors were very prominent, giving to the lips an undue fullness and a pouting expression. In order to secure room for the irregular teeth in their proper places, the right superior six-year molar, being decayed, was extracted, in preference to removing one of the bicuspids, which were in good condition. Then attempted to draw back the second right bicuspid, by means of rubber tubing attached to a clasp on the second molar, and from that stretched over the former; but six days having elapsed without any perceptible progress, a silver plate was adapted to the mouth, and clasped to the right second molar and first left bicuspid. Also interposed a gold clasp or arm against the anterior proximal surface of the right second bicuspid, and soldered it to the plate.

It is often quite difficult to confine a plate in position by clasping to the second molar, at the time when the second dentition is nearly or about completed, on account of the shortness of the crowns; for it is probable that at this time they have not, in all cases, attained to full length, so far as their eruption is concerned. Though this difficulty was present in this instance, it was overcome, and the plate rendered secure enough for the attainment of the object in view. Then, by inserting a wedge of wood between the arm of gold and the anterior proximal surface of the second bicuspid, and renewing it daily, the tooth was forced backward, while the arm or clasp prevented the wood from also acting on the first bicuspid, and driving it forward. As the second bicuspid was moved backward, the clasp, arm, or bar was readjusted against it, it being cut off, and again soldered to the plate.

Having, in less than five weeks, forced the second bicuspid, without any soreness, to occupy half of the space made vacant by the loss of the six-year molar, it was temporarily secured in position by means of very fine platinum wire fastened to it, and the clasp which was attached to the second molar.

Next, in order to move the first bicuspid, the same course was pursued as with the second, when, in about six weeks (practically two, the patient during the time having been absent nearly a month, not wearing the plate at all), space enough was obtained for the cuspidatus and lateral incisor to occupy their proper positions within the arch, the vacancy produced by the extraction of the first molar (in width four and one-half lines, or three-eighths of an inch) being reduced, by forcing back the bicuspids, to a width of one line.

After another absence of two weeks by the patient, a new plate was fitted to the mouth. Also, in order to force the cuspidatus into its proper place, a gold bar was placed across the labial surface, and the ends secured with waxed floss silk to eyes or staples soldered to the clasps on the plate.

A properly shaped piece of wood was inserted between the bar and the labial and anterior proximal surface (or labial surface and right labio-proximal angle) of the cuspidatus, and a piece of rubber tubing slipped over the former, and from that, stretched over the lateral incisor inside of the arch. A semi-circular piece of plate, soldered to the under surface of the bar, and extending against the right labio-proximal angle of the cuspidatus, tended to keep the wood in position against that and the labial surface—it being shaped to both—and new pieces of wood and ligatures adapted thereto when required. The wood not only prevented the bar from slipping, but had the effect, from the pressure produced by swelling, to force the cuspidatus inward and backward, to which was added the constant strain or tension of the rubber ligature; and thus, in the course of a month, this very firm tooth was forced inward quite a distance, and backward one and one half lines (one-eighth of an inch)

The force produced by the swelling of the wood was more effectual, because greater, than the tension of the rubber.

Subsequently, more room being required between the first bicuspid and central incisor, the width was increased to about seven lines (ten-sixteenths of an inch), the original width being five and one-half lines (seven-sixteenths of an inch). This was effected in six weeks, by again forcing the bicuspid backward, during which time, the patient was absent a month. The width of space between the cuspidatus and central incisor was originally two and one-half lines, which was increased to five.

At this time (nearly nine months, including time of absence, since the correction was commenced), the lateral incisor, though formerly shutting *inside* of the corresponding under tooth, now shut *outside*, in which position it had been for some time, having been drawn slowly forward by the action of the ligature, without the use of appliances to keep the jaws apart. Indeed, the latter is rarely, if ever, necessary. Removed the ligature, and applied force against the palatine surface, by means of a screw of silver, passing through a nut of the same material, soldered to the plate. In one day and night the screw accomplished more than the ligature did in several.

Also inserted wood between the screw and palatine surface of the lateral incisor. Subsequently, the wood and nut were used, and the screw dispensed with. Again, the patient absented himself for more than six weeks.

At the end of eleven months, including time of absence, commenced operations on the left central incisor, and at the end of eleven and one-half months (the patient having reached the age of fourteen), the teeth were all in their proper positions, all of the front teeth, with the right bicuspids, having been moved.

The left central incisor, having presented obliquely to the arch, was turned one-quarter around on its axis, in fifteen days, by the following method, though there seemed to be hardly space enough between the adjoining central and lateral incisors for the correction :

Connected rubber tubing with the staples or eyes on the clasps. Placed a gold bar, one inch in length, across the projecting angle of the central incisor, after stretching and fastening the tubing to each end, thus producing a constant pressure on the anterior labial surface or left labio-proximal angle. By means of a screw passing through a nut fastened to the plate, as in the case of the right lateral incisor, force was brought to bear against the posterior palatine surface, or right palato-proximal angle, and the tooth turned on its axis until it was regular in position, when it was secured, and the patient instructed to wear the appliances, with slight alterations to simplify them, until the teeth became firm in their new positions.

During the time occupied in the correction of the irregularity (eleven and one-half months), the patient was absent about sixteen weeks.

The teeth were much more firmly implanted in the alveoli than in most cases which are presented. Though the correction was undertaken before the right cuspidatus was fully erupted, or the left had pierced the gum—though nearly the entire crown of the latter appeared and the former finished its eruption during the time occupied in the correction—it may usually be best to await the full accomplishment of dentition, with the exception of the dentes sapientiæ, before attempting the operation, except so far as means may be employed to *prevent* their irregular presentation. That is another thing; and how to do it, is a question of much interest, and affording ample scope for serious consideration and judicious practice, in connection with the influence which the preservation and seasonable or premature removal of the deciduous teeth may have upon it.

The foregoing is not cited as a complicated case, though a stubborn one, but in order to illustrate certain effectual methods of practice. In more tractable cases, in which the arrangement of the teeth has been similar, the results have been attained to more rapidly.

For instance, where the osseous system is less perfectly developed, owing to a deficiency of lime, this may be the case; but a relatively longer time is required for the corrected teeth to become firm again. The age and condition of the patient, may also have an influence upon the progress made in the latter respect. A new growth or deposition, where the process has been absorbed or displaced by the pressure on certain portions, incident to the correction, is very likely—more tardily effected, when the osseous system has very nearly or quite reached *full* development.

In the foregoing described case, the teeth were unusually firm, and the alveoli hard and unyielding. Added to this, the patient was very robust, with a fine *physique*, and well, if not fully developed osseous system; so that, notwithstanding the length of time consumed in the correction of the irregularity, a much shorter period sufficed, after the change, for the teeth to become firm, and thus rendered the relinquishment of appliances practicable.

In the recent case of a young man, aged sixteen, with a partially irregular denture, who, when correction was commenced, had reached a height of six feet, and was still growing—and in which the left superior lateral incisor occupied a position back of the adjoining central incisor and cuspidatus, the former lapping over it, while the corresponding under tooth shut outside of it, and the left cuspidatus projected posteriorly, also lapping over the lateral incisor—the teeth were very *easily* and *rapidly* moved, by substantially the same methods as in the above described case, after obtaining the required space between the central incisor and bicuspid, by forcing the side teeth backward; but more than double the time that was employed in the correction, did not suffice to render them permanent in position.

A too rapid, and consequently imperfect or unfinished development of the osseous system—a deficient supply or lack of appropriation of the mineral elements, as compared with the relative proportions of earthy or animal matters, added to weakness or debility of the general system—may have been the cause. A too rapid growth of the latter, may indicate or carry with it a lax or unequal appropriation of all the elements which enter therein.

A general over-stimulation or over-nutrition of the body, or an increased or abnormal activity of the vital functions, and consequently a delayed, imperfect or unfinished formation of every part, and which had not settled down to the firmness, denseness, or solidity of a fully developed organism—the attainment of which might not be had until, with the cessation of growth (so far as relates to size and extent), nutrition was entirely directed thereto; these conditions may have had an influence upon the maxilla, it also sharing in the laxness and deficiency which may have characterized such a period.

At the age when only twenty-eight of the thirty-two permanent teeth have erupted, the process of active growth and development of the jaws has not ceased, and perhaps, in most cases, has not been fully accomplished until the wisdom teeth have appeared—excepting, perhaps, those where great severity is experienced in their eruption for want of room; but we find that, in such cases, the parts usually accommodate themselves to the requirements of the case, though, in many instances, very slowly.

Thus, these almost constant changes, and losses or additions of alveoli, and the organs implanted therein, give rise, more than anything else, to the variableness of expression which characterizes the individual from the period of infancy to that of senility.

It is surprising, that more is not done to remedy the evils which result from the malformation or "irregular or anomalous formation or structure of parts," as displayed in the awry, distorted and pinched expressions which are so common. Owing, in many instances, to the ignorance which exists with parents, to say nothing of cupidity in others, the remedy, though at hand, is not employed; and this at the expense of the personal appearance, comfort and health of the individual, with, in the case of thousands, an ever retrospective regret, in after life, that what might have been, is not now nor ever shall be. The fault, doubtless, lies in a great measure with many in the profession, who are averse to undertaking what, in so many instances, promises so little in the way of pecuniary reward. But this motive, though possibly defensible to some extent, ought not to be the chief and crowning one; but the more exalted and nobler, humanizing impulses, should influence and animate the soul to the carrying out of the higher conceptions emanating from a desire to honor the profession, by not only rendering it a means of usefulness, but by seeking its advancement in intelligence and virtue, as well as art, science and skill in the management of cases entrusted to our charge. The means are at hand—the end and object, though slowly reached, it may be, is attainable.

